# Bioresearch of New 1*H*-pyrrolo[3,4-c]pyridine-1,3(2*H*)-diones

**DOI:** 10.3390/molecules25245883

**Published:** 2020-12-12

**Authors:** Dominika Szkatuła, Edward Krzyżak, Szczepan Mogilski, Jacek Sapa, Barbara Filipek, Piotr Świątek

**Affiliations:** 1 Department of Medicinal Chemistry, Faculty of Pharmacy, Wroclaw Medical University, Borowska 211, 50-556 Wroclaw, Poland; piotr.swiatek@umed.wroc.pl; 2 Department of Inorganic Chemistry, Wrocław Medical University, ul. Borowska 211a, 50‑556 Wrocław, Poland; 3 Department of Pharmacodynamics, Faculty of Pharmacy, Jagiellonian University in Kraków, ul. Medyczna 9, 30-688 Kraków, Poland; szczepan.mogilski@uj.edu.pl (S.M.); jacek.sapa@uj.edu.pl (J.S.); barbara.filipek@uj.edu.pl (B.F.)

**Keywords:** 3,4-pyridinedicarboximide, analgesic and sedativeactivity

## Abstract

The subject of the work was the synthesis of new derivatives of1*H*-pyrrolo[3,4-c]pyridine-1,3(2*H*)-dione with potential analgesic and sedative activity. Eight compounds werereceived. The analgesic activity of the new compounds was confirmed in the “hot plate” test and in the “writhing” test. All tested imides **8**–**15** were more active in the “writhing” test than aspirin, and two of them, **9** and **11**, were similar to morphine. In addition, all of the new imides inhibited the locomotor activity in mice to a statistically significant extent, and two of them also prolonged the duration of thiopental sleep.On the basis of the results obtained for the previously synthesized imides and the results presented in this paper, an attempt was madeto determine the relationship between thechemical structure of imides and their analgesic and sedativeproperties.

## 1. Introduction

Pain is an important signal of the body, an element of the self-preservation instinct, but it is also a source of suffering. Despite the availability of pain medications, daily pain affects a large proportion of the world’s population and limits all aspects of a person’s life. Therefore, there is still a need to look for effective and safe tools to fight pain management, especially when current treatmentsare impossible (due to side effects or drug interactions) or ineffective. In order to improve the quality of life of patients suffering from idiopathic and chronic pain, medicine uses, among others, gene therapy ( enzyme fatty acid amide hydrolase FAAH and “pseudogene” FAHH-OUT of SCN9A gene) [1,2,3] and virtual reality (VR) methods, as well as new drug mechanisms to fight pain—e.g., small molecule prototypes of the peripheral sodium channel, NaV1.7 antagonists [3]. The search for molecules with potential analgesic activity is constantly ongoing.

So far, no mechanism for inducing an analgesic effect is known for 1*H*-pyrrolo[3,4-c]pyridine-1,3 (2*H*)-dione derivatives, but their high activity in the “writhing” test, accompanied by little or no toxicity compared to morphine and aspirin (ASA), is a good starting point for the search for an ideal antinociceptive drug [4].

**Figure 1:**
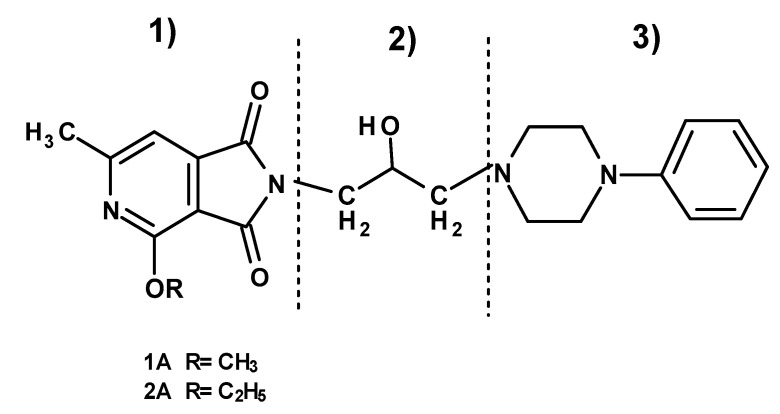
Structure of imides **1A** and **2A**. The figure shows the sections of the molecule subject to modification discussed in the text. (1) 3,4-pyridinedicarboximide, (2) linker, (3) arylamine.

The current paper presents the next stage of research on the synthesis and investigation of pharmacological properties of new 1*H*-pyrrolo[3,4-c]pyridine-1,3(2*H*)-dione derivatives. As establishedearlier, the strongest analgesic and sedative properties in the studies were observed for derivatives1A and 2A (Figure 1 and Figure S1), which were considered model compounds. They have become a point of reference to determine the relationship between the chemical structure and biological activityofpyrrolopyridine derivatives. We have obtained a series of successive derivatives, taking into account the following modifications in the structure of the basic system and the alkylarylamine linker:

(1) The type and size of the alkoxy substituent in the 2-position of pyridine, 4-alkoxy derivatives have shown stronger analgesic properties than ethoxy analogs.
(2) The role of the alkyl linker connecting the basic center of the arylamine with the cyclic imide system, 2-hydroxypropyl derivatives being the most active.
(3) The importance of pharmacophoric groups in the phenyl substituent at N-4 of the piperazine ring for the direction and strength of their biological action. Phenyl homologs, unsubstituted, and also containing electron-withdrawing groups such as −CF_3_, −F, −Cl and −OCH_3_ were obtained. In some cases, the aryl ring has been replaced by a bioisosterictetrahydroisoquinoline moiety and the piperazine ring replaced by another cyclic amine.

The results were discussedin detail in our previous works [5,6,7,8,9,10,11,12,13,14]. As previously established, the shortening of the alkyl linker between the basic center of the arylamine and the cyclic imide moiety resulted in derivatives with similar biological properties [8]. Mannich base-type imides were active in the “writhing” test, more than aspirin, were additionally endowed with sedative properties, inhibited the spontaneous locomotor activity in mice and statistically significantly extended the duration of thiopental anesthesia [8].

To determine the effect of the length of the alkyl linker connecting the arylamine with the pyrrolo[3,4-c]pyridine-1,3(2*H*)-dione ring, we decided to supplement the study with a group of N-methylene and N-ethylene derivatives, which are the subject of ongoing analysis. The methods and effects of the synthesis will be supplemented with behavioral studies. Based on the obtained results of biologicaltests, the emergingrelationshipsbetween the activity and chemicalstructure of homologsare discussed.

## 2. Results

### 2.1. Chemistry

The starting materials for the synthesis of compounds **9**–**15** were 4-methoxy- and 4-ethoxy-6-methyl-1*H*-pyrrolo[3.4-c]pyridine-1,3(2*H*)-diones(**3**,**4**), and in the case of imide **8**, it was an intermediate 4-methoxy-2,3-dihydro-6-methyl-2-(4-bromobutyl)-1,3-dioxo-1*H*-pyrrolo[3,4-c]pyridine (**5**) synthesized previously [5,8].

#### 2.1.1. N-Substituted Derivatives of 4-alkoxy-6-methyl-1H-pyrrolo[3,4-c]pyridine-1,3(2H)-diones (5, 6a, 6b, 7a, 7b)

**Figure 2:**
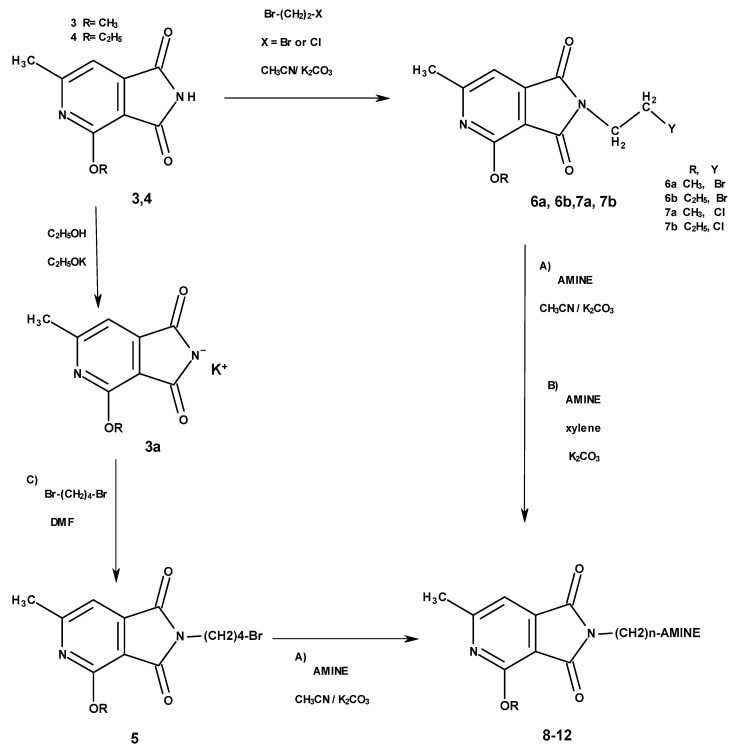
Synthesis of imides **8**–**12**.

The synthesis of the final compounds was carried out in two stages (Figure 2).

The starting reagents in the first stage of the synthesis, 4-methoxy/4-ethoxy-6-methyl-1*H*-pyrrolo[3,4-c]pyridine-1,3(2*H*)-diones [8] and 1,2-dibromoethane (**6a**,**6b**), 1-bromo-2-chloroethane (**7a**,**7b**),1,2-dibromobutane (**5**) [5], were used. The chemical structure of the obtained final products is presented in Table 1 and Figure 2.

The reaction with 1,2-dibromoethane and 1-bromo-2-chloroethane was carried out at the reflux temperature of acetonitrile against anhydrous potassium carbonate to obtain intermediate **6** and **7**, respectively.The potassium salts of 2-methoxy-6-methyl-3,4-pyridinedicarboximide (**3a**) were condensed with 1,4-dibromobutane, used in a fourfold excess, which was carried out with dimethylformamide (DMF) in room temperature, with the aim to obtain N-bromobutyl derivative **5**.

In the synthesis of intermediates **5** and **6**, a large excess of 1,2-dibromoethane and 1,4-dibromobutane, which allowed the imide substitution of only one halogen atom and a small amount of by-products (symmetrically substituted alkyl imide), wasseparated in all cases by fractional crystallization.

In the next step, N-halogenalkylimides **5**–**7**, were condensed with the appropriate cyclic amines: morpholine (**8**), phenylpiperazine (**9**,**10**), 2-methoxyphenylpiperazine (**11**,**12**).

The β-bromoethyl and γ-bromobuthyl derivative reaction was carried out again at the boiling point of acetonitrile (method A) and the β-chloroethyl derivative (method B), during heating to boiling in xylene, in both cases with anhydrous potassium carbonate.

The physicochemical properties of the N-aminoethyl finalderivatives of 3,4-pyridinedicarboximide formed on both routes (Method A and B) were identical, however, the condensation of 2-bromoethyl derivative had, as expected, higher yields, therefore, when obtaining imides **9**–**12**, this method was used.

In the case of γ-bromobutyl(**5**), in the ^1^H NMR spectrum, signals from protons were observed at the same wavelength (δ) as before [5]. Respectively: three singlets (δ = 2.62 ppm—CH_3_, 4.13 ppm—OCH_3_, 7.18 ppm—the pyridine ring proton); multiplet of methylene protons 2 x CH_2_ β and γ of butyl (δ = 1.81–1.93 ppm); two triplets of CH_2_ (δ = 3.35–3.51 ppm) and CH_2_ α (δ = 3.61–3.77ppm). The lack of a wide proton band of the N-H imide group in the low field (δ = 10.74 ppm) confirms the assumed condensation course. Their structures were confirmed by spectral (IR, ^1^H NMR) and elemental analyses (synthesis and properties of **8**–**12**).

#### 2.1.2. Synthesis of N-Aminomethyl Derivatives with Mannich Base Character (13–15)

**Figure 3:**
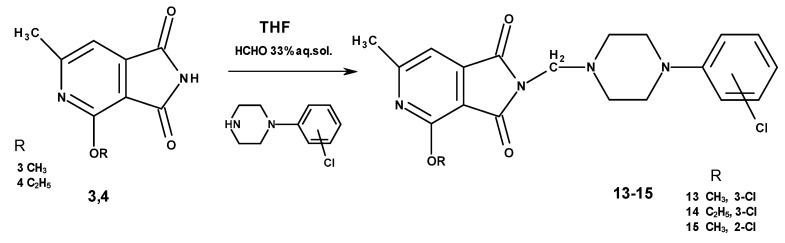
Synthesis of Mannich bases **13**–**15**.

Imides **13**–**15** weresynthesizedina Mannichreactionbyboiling 2-methoxy-/2-ethoxy-6-methyl-3,4-pyridinedicarboximide (**3**,**4**) with 33% formaline and the 2- or 3-chlorophenylo-1-piperazine in tetrahydrofuran (THF) solution (Figure 3).

### 2.2. Pharmacology

#### 2.2.1. Toxicity

The LD_50_ values of the investigated compounds after their intraperitonealadministration in mice are presented in Table 2.

Imides **8**,**10**–**15** were not toxic (LD_50_> 2000 mg/kg). One of them, imide **9**, showed a higher toxicity (LD_50_ = 1500 mg/kg). It should be noted that the analgesic efficacy of compound **9** requires the use of just 1/400th of the lethal dose or less (ED_50_ ≈ 1/408 LD_50_; Table 3). It was much safer for laboratory animals than model analgesics, aspirin or morphine, 1/40 LD_50_ or 1/57 LD_50_, respectively [16,17].

#### 2.2.2. Analgesic and Sedative Activity

In order to screen the compounds for analgesic activity, two screening methods were chosen: the “writhing” test (Table 3 and Table S1) and the “hot plate” test (Table 4).

All tested derivatives (**8**–**15**) were active in the “writhing” test (ED_50_ = 3.25–19.2 mg/kg), and their analgesic properties in this study exceeded the effect of aspirin (ED_50_ = 39.15 mg/kg). In addition, for two imides, they were similar to morphine activity (**9** = 3.25 mg/kg, **11** = 3.67 mg/kg, morphine = 2.44 mg/kg).

However, in the case of the “hot plate” test, the observed analgesic effects for the tested compounds **8**, **10**, **12**–**15** were not statistically significant. None of them had analgesic effects in the applied doses (Table 4). The derivative **9** exerted an analgesic effect in three doses: 300, 150, 75 mg/kg, and prolonged the latency time to nociceptive response by 105%, 102% and 55%, respectively. Compound **11** also prolonged the latency time by 105%, but in one 400 mg/kg dose only. For the tested derivatives, ED_50_ values were not determined, due to the non-significant effect in this test (**8**, **10**, **12**–**15)** or activity that did not fulfill the requirements for the proper calculations of the value (derivative **9** and **11**). The ED_50_ values for the reference compounds were 266.7 mg/kg (±SEM = 98.26; 148.2–533.4) and 2.55 mg/kg (± SEM = 0.63; 1.59–4.08) for ASA and morphine, respectively.

As before [5,8], the tested derivatives showed sedative properties. Imides **8**–**15** significantly inhibited spontaneous locomotor activity in mice (Table 5 and Table S2) and the two strongest (**9**, **11**) also extended the duration of thiopental anesthesia (Table 6).

The pilot biological studies for imides **9** and **11** were supplemented with the determination of the effect of the intraperitoneal administration of the test compounds on the duration of thiopental-induced sleep. The mechanism and the degree of crossing the blood–brain barrier has not been precisely defined. The observed sedative effects may indicate good penetration of compounds into the central nervous system, which, however, has not been confirmed.

## 3. Discussion

Based on our current and previous research [5,6,8,10], it is possible to determine the relationship between the structure of molecules and biological properties in the group of 3,4-pyridinedicarboximide derivatives. To this end, the impact on the animal test results of the following structural elements should be determined.

The basic modifications are: (I) an alkoxy substituent on the pyridine ring; (II) type of amino residue; (III) length of the alkyl link between these building elements.To this end, it is necessary to recall the previous conclusions (marked, to distinguish series 1 and 2), comparing the abovementioned modifications and the obtained results of experimental studies. (I) The type of alkoxy substituent on the pyridine ring has a decisive influence on the analgesic potency. The methoxyhomolog was more active in the tests. Only in halogenated derivatives was a deviation from this regularity observed. The intensification of hydrophobic properties by increasing the substituent to two carbon atoms weakened the analgesic properties, although their potential still remained noteworthy (II).

The type of amino residue, as the basic center of the molecule, is in most examples a phenylpiperazine moiety. The strongest analgesic effect was observed in derivatives with an unsubstituted benzene ring. The introduction of 2-OCH_3_/3-CF_3_ pharmacophoreic moieties and halogen (Cl/F) atoms was not preferred, similarly in the case of replacing phenylpiperazine with another cyclic amine (morpholine, tetrahydroisoquinoline).The strongest analgesic properties in the tests performed showed phenylpiperazine derivatives in each series of homologs. Only in the case of the elimination of the OH group and the propyl linker did the presence of pharmacophores in phenyl significantly “compensate” this modification. These relationships were more analgetically active. The following thesis can be adopted that the analgesic potency of 1*H*-pyrrolo [3,4-c]pyridine-1,3 (2*H*) -dione derivatives decreases according to the following series: attitudes in the benzene ring H> OCH_3_ ≥ CF_3_> Cl/F among all homologs.

A separate analysis should be done for the length of the alkyl linkage between the cyclic imide ring and the basic center of the amino residue (III). A significant influence of this element of the structure on the analgesic potency has been noticed. Compounds **1A**,**2A**, considered by us as models [6], containing an OH group in the propyl linker (racemate), showed activity similar to that noted for aspirin (ED_50_ in the “hot plate” test 10.6–96.8 mg/kg; ASA = 266.7 mg/kg) [6,8]. Not all derivatives obtained were active in this study, so we make a summary in the second, “writhing” test. In this test, the activity of the derivatives tested was similar to or higher than that of morphine (ED_50_ 0.4–2.80 mg/kg; morphine = 2.44 mg/kg, significantly higher than aspirin = 39.15 mg/kg, Table 7).

Elimination of the OH group weakened the analgesic effect. The ED_50_ of propyl derivatives was 0.67–1.03–1.10–2.59 mg/kg, respectively.

As mentioned, compounds containing the 2-OCH_3_ pharmacophore in phenyl were more active. With the shortening of the distance to C-2 and C-1, activity decreased. Additionally, elongation of the C-4 linker (butyl derivatives) did not increase analgesic activity. To illustrate the above conclusions, Table 7 summarizes ED_50_ values in the “writhing” test in mice of phenylpiperazine analogs (**1H**, **2H**, **9**, **1K**), 2-methoxyphenylpiperazine analogs (**1I**, **1P**, **11**, **1L**) and all amines (**1N**, **1M**, **8**, **1Q**) or halogens (**1J**, **2J**) in Table 8 [10].

Modifications to the structure of the model **1A** imide resulted in the occurrence of a calming effect in the test, determining the effect of the compounds tested on the spontaneous mouse mobility. Some of the derivatives obtained were additionally tested in a thiopental test.

All derivatives, obtained as a result of the modification of the imide structure **1A**, statistically significantly inhibited the spontaneous locomotion activity of mice. The ED_50_ values of homologs are given in Table 9. Analyzing the relationship between the chemical structure of the molecule and the sedative activity of homologs, one can notice a large variation, depending on all introduced modifications. The strongest sedative properties were observed in the case of imide **1K** (Mannich base character), while the effective dose values of other derivatives from the same group were much higher.

It is not possible to identify elements of the structure clearly responsible for the sedative effect. The results in Table 9 illustrate the significant variation in CNS inhibitory properties for the listed derivatives.

The statistically significant analgesic activity of 3,4-pyridinedicarboximide derivatives, discussed above, does not allow for determining the mechanism of the analgesic activity of new compounds and its relationship with the sedative effect. One direction of our further research was a pilot determination of the affinity of some imides for μ opioid receptors. To this end, our team performed an experiment to determine the ligand displacement ability of tritium-labeled dihydromorphine [^3^H-DHM] from binding sites of the µ receptor of the rat cerebral cortex (Table S3).Selected derivatives displaced the ligand at a concentration significantly exceeding 100 nM. Morphine and tramadol (a centrally acting synthetic opioid analgesic and serotonin/norepinephrine reuptakeinhibitor (SNRI), much simpler than opioids) have receptor affinity at concentrations several times lower: 0.62 and 2.4 nM, respectively [18,19,20]. It can be concluded that the new derivatives that we synthesized and tested may became a new and useful class of analgesics with a unique mechanism of action, including cyclooxygenase(COX) inhibition at the lower doses and opioid receptor activation at higher ones. For this purpose, further studies will be needed to determine the affinity of 3,4-pyridinedicarboximides for the enzymes of the arachidonic acid pathway and for opioid receptors.

## 4. Materials and Methods

### 4.1. Chemistry

All the results of the C,H and N determinations (carried out by a Carlo Erba Elemental Analyzer model NA-1500, Carlo Erba, Thermo Scientific, Waltham, MA, USA) were within ± 0.4% of the theoretical values. All melting points are uncorrected. The IR spectra, in KBr pellets, were measured with a Zeiss Jena Specord model IR75 (Zeiss Jena, Uberlingen, Germany) and^1^H NMR spectra were determined in CDCl_3_, if not otherwise indicated, on a Tesla 587 A spectrometer (80 MHz, Tesla, Brno, Czech Republic) using tetramethylsilane (TMS) as an internal standard.

#### 4.1.1. Procedure for Obtaining 4-methoxy-6-methyl-4-(N-morpholino)-butyl-1H-pyrrolo[3,4-c]pyridine-1,3-(2H)-dion(8)

To 0.003 mol of compound **3** in 70 mL of anhydrous acetonitrile, 0.75 g of anhydrous potassium carbonate and 0.004 mol morpholine were added. The mixture was refluxed for 21h. After filtration, the solvent was evaporated under reduced pressure and the residue was purified by crystallization from ethanol.

The properties of **8**:Formula: C_17_H_23_N_3_O_3_; MW = 333.38; MP: 92–93 °C; solvent: ethanol; yield: 51%; IR (cm^−1^): C=O 1720, 1780; -CH_2_- 2800–2980;^1^H NMR of 8: δ [ppm] = 1.34–2.22 (m-4H. Hβ + Hγ of butyl); 2.25–2.70 (m-9H. CH_3_ + -CH_2_-N(CH_2_)_2_); 2.34–4.20 (m-6H. Hα of butyl + -(CH_2_)_2_O); 4.13 (s-3H. OCH_3_); 7.18 (s-1H. H of pyridine).

#### 4.1.2. Procedure for Obtaining 4-methoxy-/4-ethoxy-6-methyl-2-(2-bromoethylo)-1H-pyrrolo-[3,4-c]pyridine-1,3(2H)-diones (6a, 6b)

To 0.01mol of compound **3** in 70 mL of anhydrous acetonitrile, 0.01 mol of anhydrous potassium carbonate were refluxed for 0.5 h. Next, 0.04 mol 1,2-dibromoethan were added. The mixture was refluxed for 17 h. After hot filtration, the inorganic material was washed with acetonitrile. The solvent was completely evaporated on a rotary evaporator. The dry residue was crystallized from n-hexane (**6a**), or cyclohexane (**6b**) to give a light yellow amorphous product.The physicochemical properties are given in Table 10.

^1^H NMR of **6a**: δ [ppm] = 2.62 (s-3H, CH_3_); 3.34–3.74 (t-2H, Hβ of ethyl); 3.84–4.28 (m-5H, OCH_3_ + Hα of ethyl); 7.11 (s-1H, H of pyridine).

^1^H NMR of **6b**: δ [ppm] = 1.32–1.65 (t-3H, -OCH_2_CH_3_); 2.61 (s-3H, CH_3_); 3.47–3.71 (t-2H, Hβ of ethyl); 3.92–4.23 (t-2H, Hα of ethyl); 4.42–4.78 (q-2H, -OCH_2_CH_3_); 7.17 (s-1H, H of pyridine).

#### 4.1.3. Procedure for Obtaining 4-methoxy-/4-ethoxy-6-methyl-2-(2-chloroethylo)-1H-pyrrolo[3,4-c]pyridine-1,3(2H)-diones (7a, 7b)

To 0.01 mol of compound **3** in 70 mL of anhydrous acetonitrile, 0.01 mol of anhydrous potassium carbonate were refluxed for 0.5 h. Next, 0.01 mol 1-bromo-2-chloroethan were added. The mixture was refluxed for 17 h. After hot filtration, the inorganic material was washed with acetonitrile. The solvent was completely evaporated on a rotary evaporator. The dry residue was crystallized from cyclohexane (**7a**,**7b**) to give a light yellow amorphous product.The physicochemical properties are given in Table 10.

^1^H NMR of **7a**: δ [ppm] = 2.63 (s-3H, CH_3_); 3.56–4.06 (m-4H, Hα + Hβ of ethyl); 4.13 (s-3H, OCH_3_); 7.20 (s-1H, H of pyridine).

^1^H NMR of **7b**: δ [ppm] = 1.31–1.78 (t-3H, -OCH_2_CH_3_); 2.61 (s-3H, CH_3_); 3.75–3.86 (t-2H, Hα of ethyl); 3.87–4.18 (t-2H, Hβ of ethyl); 4.24–4.84 (m-2H, -OCH_2_CH_3_); 7.18 (s-1H, H of pyridine).

#### 4.1.4. General Procedure for Obtaining Compounds 9–12

Method A

To 0.01 mol of compound **6a** or **6b** and 0.01 mol of anhydrous potassium carbonate, 70 mL of acetonitrile and 0.02 mol of suitable amine (4-phenyl-1-piperazine; 4-(2-methoxyphenyl-1-piperazine) in 10 mL of acetonitrile were added. The mixture was refluxed for 17 h. After hot filtration (simple paper filter), the inorganic material was washed with acetonitrile. The solvent was completely evaporated on a rotary evaporator. The dry residue was crystallized from cyclohexane to give a light yellow product.

The physicochemical properties of compounds **9**–**12** are given in Table 10.

Method B

To 0.01 mol of the compound, suspended in 40 mL of dried xylene, 0.01 mol of anhydrous potassium carbonate and 0.02 mol of the corresponding amine were added. The whole mixture was heated to reflux with constant stirring for 15 h. Then it was filtered and the inorganics were washed with solvent and evaporated completely. The resulting oil was crystallized from cyclohexane or a mixture with ethanol (properties of compounds **9**–**12** in Table 10, method B).

^1^H NMR of **9**: δ [ppm] = 2.34–2.86 (m-9H, CH_3_ + -CH_2_-N-(CH_2_)_2_-); 2.88–3.27 (m-4H, -(CH_2_)_2_-N-); 3.62–3.94 (t-2H, Hα of ethyl); 4.11 (s-3H, OCH_3_); 6.62–7.40 (m-6H, H arom.).

^1^H NMR of **10**: δ [ppm] =1.13–1.63(t-3H, -OCH_2_CH_3_); 2.21–2.86 (m-9H, CH_3_ + -CH_2_-N(CH_2_)_2_-); 3.05–3.51 (distorted t-4H, H-piperazine); 3.64–4.02 (t-2H, Hα of ethyl); 4.37–4.84 (q-2H, -OCH_2_CH_3_); 6.59–7.47 (m-6H, H arom.).

^1^H NMR of **11**: δ [ppm] = 2.54–2.88 (m-9H, CH_3_ + -CH_2_-N-(CH_2_)_2_-); 2.88–3.16 (m-4H, -(CH_2_)_2_-N-); 3.73–3.98 (m-5H. OCH_3_ + Hα of ethyl); 4.13 (s-3H, OCH_3_); 6.73–7.38 (m-5H, H arom.).

^1^H NMR of **12**: δ [ppm] =1.27–1.62 (t-3H, -OCH_2_CH_3_); 2.44–2.84 (m-9H, CH_3_ + -CH_2_-N(CH_2_)_2_-); 2.90–2.96 (m-4H, H-piperazine); 3.34–4.01 (m-5H, -OCH_3_+Hα of ethyl); 4.38–4.75 (q-2H,-OCH_2_CH_3_); 6.72–7.33 (m-5H, H arom.).

#### 4.1.5. General Procedure for Obtaining Compounds 13–15

To 0.002 mole of 2-methoxy or 2-ethoxy-6-methyl-3,4-pyridinedicarboximide (**3**,**4**), suspended in 40 mL tetrahydrofuran, 1 ml 33% formalin was added. The reaction mixture was heated for 0.5 h, then 0.0022 mol of the corresponding amine (2- or 3-chloro-4-phenyl-1-piperazine) were added again to reflux for 10 h. The whole mixture was evaporated completely on a rotary evaporator and the remaining oil crystallized from suitable solvents (Table 10).

The following is the interpretation of ^1^H NMR spectra of compounds **13**–**15**.

^1^H NMR of **13**: δ [ppm] = 2.63 (s-3H, CH_3_); 2.67–2.86 (t-4H, -N-(CH_2_)_2_-); 3.00–3.26 (t-4H, -(CH_2_)_2_-N-); 4.13 (s-3H, OCH_3_); 4.66 (s-2H, -CH_2_-); 6.66–7.27 (m-5H, H arom.).

^1^H NMR of **14**: δ [ppm] =1.45–1.55 (t-3H, -OCH_2_CH_3_); 2.62 (s-3H, CH_3_); 2.73–2.95 (t-4H, -N(CH_2_)_2_-); 3.15–3.35 (t-4H, H-piperazine); 4.59–4.78 (m-4H, -CH_2_- + -OCH_2_CH_3_); 6.70–7.25 (m-5H, H arom.).

^1^H NMR of **15**: δ[ppm] = 2.64 (s-3H, CH_3_); 2.73–2.86 (t-4H, -N-(CH_2_)_2_-); 3.06–3.36 (t-4H, -(CH_2_)_2_-N-); 4.14 (s-3H, OCH_3_); 4.68 (s-2H, -CH_2_-); 6.88–7.43 (m-5H, H arom.).

### 4.2. Materials and Methods of Pharmacology Experiments

#### 4.2.1. Substances

Acetylicacid (polopiryna, Polpharma, Starogard Gdański, Poland). Morphine (morphinum hydrochloridum, Polfa-Kutno, Poland).

#### 4.2.2. Animals

The experiments were carried out on male albino Swiss mice (body weight 18–26 g). All of the animals were housed at constant humidity (60%) and temperature (25 °C) and kept on a 12 h light/dark cycle. Animals were fed a standard pellet diet with free access to tap water. All procedures were conducted according to Animal Care and Use Committee guidelines, and approved by the Ethical Committee of Jagiellonian University, Kraków.

Control and experimental groups consisted of 6–8 animals each. The investigated compounds were administered intraperitoneally as a suspension in 0.5% methylcellulose in a constant volume of 10 mL/kg.

### 4.3. Statistical Analysis

The statistical significance was calculated using a Student’s *t*-test. The ED_50_ values and their confidence limits were calculated according to the method of Litchfield and Wilcoxon [8,15].

The obtained 95% confidence limits were transformed to standard errors of the mean (SEM), as described previously [21].

### 4.4. Acute Toxicity

Acute toxicity was assessed by the methods of Litchfield and Wilcoxon [8,16]and presented as LD_50_ calculated from the mortality of mice after 24 h.

### 4.5. Pain Reactivity

Pain reactivity was measured in two tests: “hot plate” test (according to the method of Eddy and Leimbach [9,22]) and “writhing” test in mice (according to Hendershot and Forsaith [8,23]).

#### 4.5.1. “Hot plate” Test

Animals were placed individually on the metal plate, heated to 55 ± 1 °C. The latency time (s) to the pain reaction (licking of the hind paws or jumping) was recorded by a stop-watch. A cutoff time of 45 s was used to prevent tissue damage. The experiment was performed 30 min after the administration of the investigated compounds at graded doses of 4.5–100 mg/kg (1/160–1/20 LD_50_i.p.).

#### 4.5.2. “Writhing” Test in Mice

Different doses of the compounds, ranging from 0.39 to 100 mg/kg (1/5120–1/20 LD_50_i.p.), were administered intraperitoneally. Then, after 25 min, the irritant (phenylbenzoquinone, 0.02% solution, ethanol-water, 5:95) was also administered intraperitoneally in a constant volume of 0.25 mL. Five min after the application of the irritant,a 10 min long period of observation started, in which the number of writhing episodes was counted.

The analgesic effect of individual doses was expressed in percent:

(1) $$\%\;\mathrm{Analgesic}\;\mathrm{effect}=100-\frac{\sum \;\mathrm{of}\;\mathrm{writing}\;\mathrm{incidents}\;\mathrm{in}\;\mathrm{experimental}\;\mathrm{group}}{\sum \;\mathrm{of}\;\mathrm{writhing}\;\mathrm{incidents}\;\mathrm{in}\;\mathrm{control}\;\mathrm{group}}\times 100$$

### 4.6. Sedative Effect

Spontaneous locomotor activity in mice was measured in circular photoresistoractometers (32 cm in diameter). The investigated compounds were injected intraperitoneally, at a dose range of 1.56–50 mg/kg. Thirty minutes after the injection of the investigated compounds, mice were placed in the actometers for 30 min. Each crossing of the light beam was recorded automatically. The amount of impulses was noted after 30 min.

## 5. Conclusions

The values of ED_50_ in the spontaneous locomotor activity test are significantly higher than those obtained in the “writhing” test (the values were 5- and 6-fold higher for derivatives **9** and **11**, respectively). These results show that the sedative effect of the tested compounds does not affect the analgesic effect in the “writhing” test. On the other hand, the analgesic effect in the “hot plate” test may result from the sedative activity of the compounds or may be in line with that activity. As it can be noticed for morphine, opioids are active in both tests within a similar range of doses, whereas drugs mainly active in the inflammatory pain, such as Nonsteroidal anti-inflammatory drugs NSAIDs (including aspirin), are active in the “writhing” test, while being much less active in the “hot plate” test. The results for the test compounds show that they are active in chemogenic inflammatory pain rather than in acute pain induced by a thermal stimulus. Thus, their pharmacological profile resembles the activity of NSAIDs and suggests that their mechanism of action may result from the inhibition of cyclooxygenase function. To confirm or exclude this hypothesis, some further biochemical studies of affinity for cyclooxygenase (COX) and opioid receptors should be performed. On this basis, it will be possible to determine the correlation between the effect confirmed in tests and the results of theoretical molecular docking.

## Figures and Tables

**Table 1:** Structure of imides **8**–**12**.

Compound	R	n	Amine	Compound	R	n	Amine
**8**	CH_3_	4	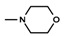	**11**	CH_3_	2	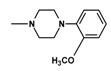
**9**	CH_3_	2	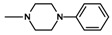	**12**	C_2_H_5_	2	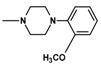
**10**	C_2_H_5_	2	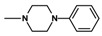				

**Table 2:** Acute toxicity of test compounds and Aspirin (ASA) and Morphine after intraperitoneal administration according to Litchfield and Wilcoxon [15].

**Compound**	**LD_50_(mg/kg)**
**8**	>2000
**9**	1500 (1395.0–1710.0)
**10**–**15**	>2000
**ASA [15]**	167.0
**Morphine [16]**	140.0

The data are median lethal doses with 5 % confidence limits in parentheses (n = 6).

**Table 3:**
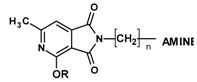
Influence of the compounds investigated on the pain reaction in the “writhing” test in mice.

Compounds	R	n	Amine	ED_50_ (mg/kg) ± SEM
**8**	CH_3_	4	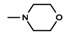	14.5 ± 0.03 (11.15–11.28)
**9**	CH_3_	2	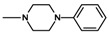	3.67 ± 0.49 (2.82–4.77)
**10**	C_2_H_5_	2	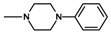	15.8 ± 0.91 (14.1–17.7)
**11**	CH_3_	2	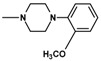	3.25 ± 0.80 (2.01–5.16)
**12**	C_2_H_5_	2	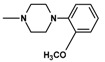	14.9 ± 2.01 (11.5–19.4)
**13**	CH_3_	1	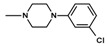	14.8 ± 1.40 (12.4–17.9)
**14**	C_2_H_5_	1	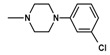	18.4 ± 1.73 (15.3–22.1)
**15**	CH_3_	1	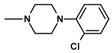	19.2 ± 2.14 (14.3–22.7)
**ASA**				39.15 ± 4.84 (29.1–48.1)
**Morphine**				2.44 ± 0.97 (1.18–5.02)

**Table 4:** Influence of the compounds investigated on the pain reaction in the “hot plate” test in mice.

Compounds	Dose (mg/kg)	Prolonged Time (%)	Time of Reaction to Pain Stimulus (s) ± SEM
Control	0		9.57 ± 1.8
**8**	200	27.48	12.2 ± 1.4
	100	3.87	10.2 ± 1.8
Control	0		19.5 ± 2.6
**9**	300	105.1 ****	40.0 ± 4.4 ****
	150	101.0 ***	39.2 ± 5 ***
	75	55.38 *	30.3 ± 2.7 *
Control	0		17.2 ± 2.1
**10**	200	34.88	23.2 ± 2.6
	100	6.98	18.4 ± 1.7
Control	0		19.5 ± 2.6
**11**	400	105.1 **	40.0 ± 8.5 **
	200	50.7	29.4 ± 5.0
	100	23.0	24.0 ± 5.0
Control	0		17.2 ± 2.1
**12**	200	52.33	26.2 ± 3.1
	100	28.46	22.1 ± 2.7
Control	0		17.2 ± 2.1
**13**	200	11.62	19.2 ± 1.8
	100	4.07	17.9 ± 2.4
**14**	200	16.82	20.1 ± 2.3
	100	2.32	17.6 ± 2.4
**15**	200	15.69	19.9 ± 3.9
	100		17.0 ± 2.8
Control	0		14.5 ± 3.6
**ASA**	400	115.86 **	31.3 ± 1.2 **
	200	35.17	19.6 ± 4.1
	100	11.72	16.2 ± 4.9
**Morphine**	6	111.10 **	30.6 ± 3.9 **
	3	104.13 *	29.6 ± 6 *
	1	33.79	19.4 ± 2.1

Each group consisted of six to eight animals. **** *p* < 0.001, *** *p* < 0.01, ** *p* < 0.02. * *p* < 0.05.

**Table 5:**
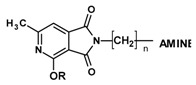
Influence of the compounds investigated on the spontaneous locomotor activity in mice.

Compounds	R	n	Amine	ED_50_ (mg/kg) ± SEM
**8**	CH_3_	4	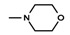	34.2 ± 8.50 (21.37–54.72)
**9**	CH_3_	2	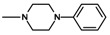	18.8 ± 4.00 (12.5–28.2)
**10**	C_2_H_5_	2	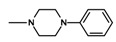	84.0 ± 5.10 (75–95)
**11**	CH_3_	2	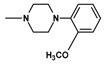	19.7 ± 4.89 (12.3 – 31.5)
**12**	C_2_H_5_	2	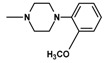	85.0 ± 4.20 (77–93.5)
**13**	CH_3_	1	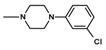	164.0 ± 28.72 (117–229.6)
**14**	C_2_H_5_	1	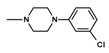	98.0 ± 13.26 (75.4–127.4)
**15**	CH_3_	1	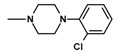	89.1 ± 4.46 (80–97.5)

**Table 6:** Influence of the compounds investigated on thiopental anesthesia.

Compounds	Dose (mg/kg)	Prolongation (%)	Duration of Anesthesia ± SEM (min)
Control	0		51.5 ±11.2
**9**	37.5	157.1 **	132.4 ± 27.8 **
	18.75	96.5 *	101.2 ± 28.4 *
	9.375	34.8	69.4 ± 12
**11**	50	140.8 ***	124 ± 16.2 ***
	25	16.11 **	50.8 ± 14.2 **
	12.5	36.3	70.2 ± 24

Each group consisted of six to eight animals. *** *p* < 0.01, **p < 0.02. * *p* < 0.05.

**Table 7:**
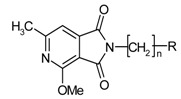
Influence of the 2-methoxy homologs on the pain reaction in the “writhing” test in mice/compounds obtained previously (series **1** and **2**) [5,6,8,10] and new imides (**8**,**9**).

Compound	R	n	ED_50_ (mg/kg)
**1H**	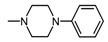	3	1.03
**1P**	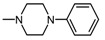	4	4.5
**9**	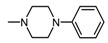	2	3.67
**1K**	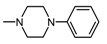	1	2.55
**1I**	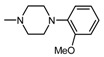	3	0.67
**1R**	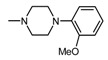	4	6.8
**11**	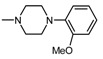	2	3.25
**1L**	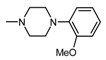	1	6.53
**1N**	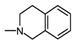	4	0.72
**1M**	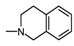	1	12.7
**8**	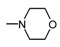	4	1.5
**1Q**	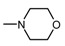	1	13.66

**Table 8:**
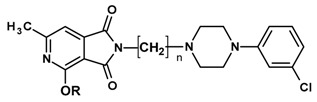
Influence of the halogen homologs on the pain reaction in the “writhing” test in mice, imides described previously (**1J**, **2J**) and new imides **13**,**14 [10]**.

Comp.	R	n	ED_50_ (mg/kg)
**1J**	CH_3_	3	8.8
**2J**	C_2_H_5_	3	8.7
**13**	CH_3_	1	14.8
**14**	C_2_H_5_	1	18.4

**Table 9:**
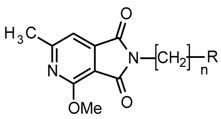
Comparison of sedative properties of selected homolog imides described previously (series **1**) [5,6,8] and new derivatives **8**,**9**,**11**.

Compound	R, n	Influence on the Locomotor Activity			Thiopental Anesthesia	
		ED_50_ (mg/kg)	Dose (mg/kg)	% Inh.	Dose (mg/kg)	% Prolong.
**1H**	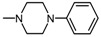 , 3	15.17	50 25 12.5	66.55 *** 53.84 ** 47.67 *	50 25	194 *** 118 *
**1P**	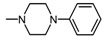 , 4	-	100 50	86.10 ** 67.14 *	100 50	242.13 *** 140.25 ***
**9**	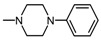 , 2	18.8	37.5 18.75 9.37	67.18 *** 45.90 ** 42.79 *	37.5 18.75	157.1 ** 96.5*
**1K**	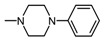 , 1	2.28	4.5 2.25	61.77 *** 49.23 **	4.5 2.25	199.1 *** 134.1 *
**1I**	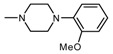 3	11.8	50 25 12.5	76.69 **** 72.77 **** 53.91 **	50 25 12.5	207.2 ** 140.3 * 123.3 *
**1R**	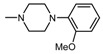 , 4	-	100	82.32 **	100 50	255.97 *** 159.12 ***
**11**	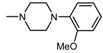 , 2	19.7	50 25 12.5	68.74 *** 55.85 ** 55.43 *	50 25	140.8 *** 16.11 **
**1L**	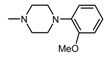 , 1	25.2	50 25 12.5	68.25 *** 40.84 * 37.81 *	50 25 12.5	184.3 *** 133.2 ** 111.6 *
**1N**	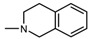 , 4		46	87.48 *	46 23	286.5 *** 100.0 *
**1M**	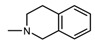 , 1	29.5	50 25	59.21 *** 47.25 **	50 25	195.1 *** 146.6 **
**8**	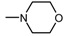 , 4	34.2	50 25 12.5	61.64 **** 35.36 ** 32.76 **	-	Not tested
**1Q**	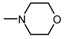 , 1		44	42.65 *	44	141.7 **

Each group consisted of 6-8 animals. **** *p* < 0.001, *** *p* < 0.01, ** *p* < 0.02, * *p* < 0.05.

**Table 10:** Physical data of 3,4-pyridinedicarboximides **6**–**13**.

Compound	Formula (mol. wt.)	M.p. (°C) Solvent	Yield (%)/Method	IR Absorptions in KBr (cm^−1^)		
				C=O	CH_2_	Mono- and Disubst. Benzene
**6a**	C_11_H_11_BrN_2_O_3_ 299.13	113–114 n-hexane	42	1718 1771	2920 2950	-
**6b**	C_12_H_13_BrN_2_O_3_ 313.10	100–102 Cyclohexane	45	1720 1770	2900 2950	-
**7a**	C_11_H_11_ClN_2_O_3_ 254.67	110–112 Cyclohexane	79	1740 1770	2920 2950	-
**7b**	C_12_H_13_ClN_2_O_3_ 268.69	102–103 Cyclohexane	81	1730 1780	2900 2980	-
**8**	C_17_H_23_N_3_O_4_ 333.38	92–93 ethanol	51	1720 1780	2800 2980	-
**9**	C_21_H_24_N_4_O_3_ 380.43	150–152 Cyclohexane	51.7/A 29/B	1717 1770	2820 2950	690,756
**10**	C_22_H_26_N_4_O_3_ 394.47	118–120 Cyclohexane	55/A 35/B	1715 1770	2840 2940	690,750
**11**	C_22_H_26_N_4_O_4_ 410.46	175–176 Cyclohexane	48.5/A 32/B	1714 1769	2930 2950	748
**12**	C_23_H_28_N_4_O_4_ 424.50	165–167 Ethanol/cyhlohexane	55/A 37/B	1715 1765	2820 2940	750
**13**	C_20_H_21_ClN_4_O_3_ 400.5	157–160 n-heptane	65	1720 1770	-	690,750
**14**	C_21_H_23_ClN_4_O_3_ 414.50	127–129 n-heptane	70	1715 1775	-	695,750
**15**	C_20_H_21_ClN_4_O_3_ 400.5	162–164 n-heptane	52	1720 1770	-	750

## Supplementary Materials

The following materials are available online. Figure S1. Structure of 1*H*-pyrollo[3,4-c]pyridine-1,3(2*H*)-dione derivatives previously published and discussed in the text (**1A**–**F**, **2A**–**F**). Part I. Figure S2. Structure of 1*H*-pyrollo[3,4-c]pyridine-1,3(2*H*)-dione erivatives previously published and discussed in the text (**1H**–**R**, **2H**–**J**). Part II. Table S1. Detailed data of the influence of the compounds investigated in the pain reaction in the “writhing” test in mice. Table S3. Detailed data of the influence of the compounds investigated in the spontaneous locomotor activity in mice. Table S2. Determination of the ability to displace ligands labeled with tritium dihydromorphine [^3^H-DHM] from the µ receptor binding sites of the rat cortex.

## References

[1] Palmer R.H.C., McGeary J.E., Knopik V.S., Bidwell L.C., Metrik J.M. (2019). CNR1 and FAAH variation and affective states induced by marijuana smoking. Am. J. Drug Alcohol Abus..

[2] Bang S., Yoo J., Gong X., Liu D., Han Q., Luo X., Chang W., Chen G., Im S.-T., Kim Y.H. (2018). Differential Inhibition of Nav1.7 and Neuropathic Pain by Hybridoma-Produced and Recombinant Monoclonal Antibodies that Target Nav1.7: Differential Activities of Nav1.7-Targeting Monoclonal Antibodies. Neurosci. Bull..

[3] Chew L.A., Bellampalli S.S., Dustrude E.T., Khanna R. (2019). Mining the Nav1.7 interactome: Opportunities for chronic pain therapeutics. Biochem. Pharmacol..

[4] Dziubina A., Szkatuła D., Gdula-Argasińska J., Kotańska M., Filipek B. (2019). Antinociceptive, antiedematous, and antiallodynic activity of 1H-pyrrolo[3,4-c]pyridine-1,3(2H)-dione derivatives in experimental models of pain. Naunyn-Schmiedeberg’s Arch. Pharmacol..

[5] Sladowska H., Szkatula D., Filipek B., Maciag D., Sapa J., Zygmunt M. (2001). ChemInform Abstract: Synthesis and Properties of 2-(4-Substituted)butyl Derivatives of Some 2,3-Dihydro-1,3-dioxo-1H-pyrrolo[3,4-c]pyridines. Chemin.

[6] Śladowska H., Filipek B., Szkatuła D., Sabiniarz A., Kardasz M., Potoczek J., Sieklucka-Dziuba M., Rajtar G., Kleinrok Z., Lis T. (2002). Investigations on the synthesis and pharmacological properties of 4-alkoxy-2-[2-hydroxy-3-(4-aryl-1-piperazinyl)propyl]-6-methyl-1H-pyrrolo[3,4-c]pyridine-1,3(2H)-diones. Farmaco.

[7] Muszalska I., Śadowska H., Szkatuła D. (2003). A validated spectrophotometric and liquid chromatography method for determination and purity evaluation of 4-methoxy-2-[2-hydroxy-3(4-phenyl-1-piperazinyl)]propyl-2,3-dihydro-6-methyl-1,3-dioxo-1H-pyrrolo[3,4-c]pyridine. Farmaco.

[8] Sladowska H., Filipek B., Szkatuła D., Sapa J., Bednarski M., Ciołkowska M. (2005). Investigations on the synthesis and pharmacological properties of N-substituted derivatives of 4-alkoxy-6-methyl-1H-pyrrolo[3,4-c]pyridine-1,3(2H)-diones. Farmaco.

[9] Muszalska I., Śladowska H., Szkatuła D. (2005). Quantitative Determination of 4-Ethoxy-2-[2-Hydroxy -3-(4-Phenyl-1-Piperazinyl)]-Propyl -2,3-Dihydro-6-Methyl-1,3-Dioxo-1H-Pyrrolo-[3,4-c] Pyridine Applying High-Performance Liquid Chromatography Using UV Detection. Studies on Degradation Mechanism. Chem. Anal..

[10] Śladowska H., Sabiniarz A., Szkatuła D., Filipek B., Sapa J. (2007). Synthesis and properties of 4-alkoxy-2-[2-hydroxy-3-(4-o,m,p-halogenoaryl-1 -piperazinyl)propyl]-6-methyl-1H-pyrrolo-[3,4-c]pyridine-1,3(2H)-diones with analgesic and sedative activities. Acta Pol. Pharm. -Drug Res..

[11] Muszalska I., Górski P., Sladowska H., Szkatuła D., Sabiniarz A. (2007). Chromatographic Separation of Derivatives of 4?Alkoxy?6?methyl?1 H ?pyrrolo[3,4?c]pyridine?1,3(2H)?dione by TLC and HPLC. J. Liq. Chromatogr. Relat. Technol..

[12] Krzyżak E., Szkatuła D., Szczęśniak-Sięga B.M., Malinka W. (2014). Synthesis and DSC study a new pyridinedicarboximide diones derivatives, obtained under various conditions. J. Therm. Anal. Calorim..

[13] Muszalska I., Ciemniejewski M.P., Lesniewska M.A., Szkatuła D., Malinka W. (2015). Forced Degradation and Photodegradation Studies of Pyrrolo[3,4-c]pyridine-1,3-dione Derivatives as Analgesic Active Compounds Using HPLC, UV and IR Spectrometry, and HPLC/MS Methods. J. AOAC Int..

[14] Krzyżak E., Szkatuła D., Wiatrak B., Gębarowski T., Marciniak A. (2020). Synthesis, Cyclooxygenases Inhibition Activities and Interactions with BSA of *N*-substituted 1*H*-pyrrolo[3,4-c]pyridine-1,3(*2H*)-diones Derivatives. Molecules.

[15] Litchfield J.T., Wilcoxon F. (1949). A simplified method of evaluating dose-effect experiments. J. Pharmacol. Exp. Ther..

[16] United States Patent Document. Vol. #4205173. htpp://chem.sis.nlm.nih.gov/chemidplus/[morphine].

[17] Kleemann A., Engel J., Kutscher B., Reichert D. (2009). Pharmaceutical Substances, 5th Edition, 2009: Syntheses, Patents and Applications of the Most Relevant APIs.

[18] Hennies H.H., Friderichs E., Schneider J. (1988). Receptor binding, analgesic and antitussive potency of tramadol and other selected opioids. Arzneimittelforschung.

[19] Raffa R.B., Friderichs E., Reimann W., Shank R.P., E Codd E., Vaught J.L. (1992). Opioid and nonopioid components independently contribute to the mechanism of action of tramadol, an ’atypical’ opioid analgesic. J. Pharmacol. Exp. Ther..

[20] Gillen C., Haurand M., Kobelt D.J., Wnendt S. (2000). Affinity, potency and efficacy of tramadol and its metabolites at the cloned human µ-opioid receptor. Naunyn-Schmiedeberg’s Arch. Pharmacol..

[21] Luszczki J.J., Borowicz K.K., Swiader M., Czuczwar S.J. (2003). Interactions Between Oxcarbazepine and Conventional Antiepileptic Drugs in the Maximal Electroshock Test in Mice: An Isobolographic Analysis. Epilepsia.

[22] Eddy N.B., Leimbach D. (1953). Synthetic Analgesics. II. Dithienylbutenyl- and Dithienylbutylamines. J. Pharmacol. Exp. Ther..

[23] Hendershot L.C., Forsaith J. (1959). Antagonism of the frequency of phenylquinone-induced writhing in the mouse by weak analgesics and nonanalgesics. J. Pharmacol. Exp. Ther..

