# Supplementary material for: Bioresearch of New 1H-pyrrolo[3,4-c]pyridine-1,3(2H)-diones
Source: Molecules. 2020 Dec 12;25(24):5883. doi: 10.3390/molecules25245883 (PMC7764601; doi:10.3390/molecules25245883)
Supplement: Supplementary file 1 [file molecules-25-05883-s001.pdf]

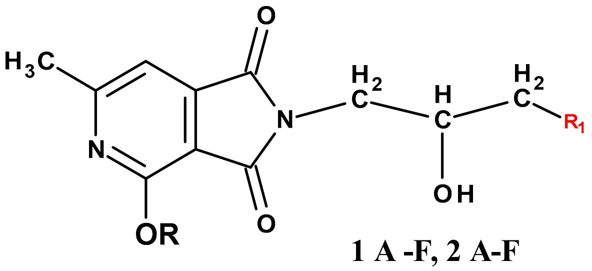

1 A -F, 2 A-F

**Comp.**	**R**	**R1**	**Comp.**	**R**	**R1**
**1A**	CH_3	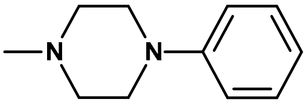	**2A**	C_2H_5	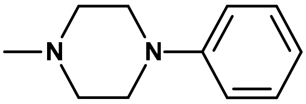
**1B**	CH_3	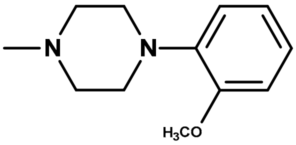	**2B**	C_2H_5	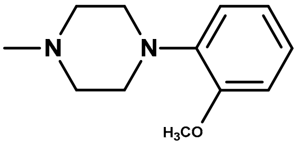
**1C**	CH_3	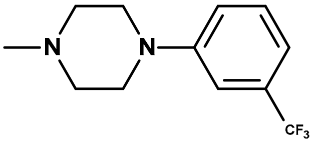	**2C**	C_2H_5	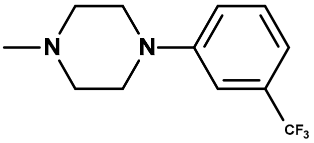
**1D**	CH_3	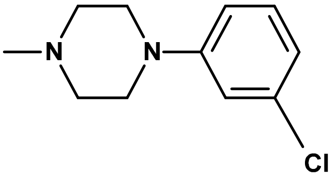	**2D**	C_2H_5	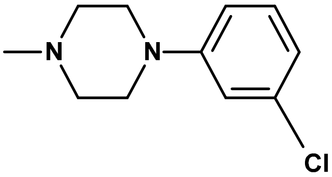
**1E**	CH_3	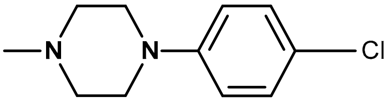	**2E**	C_2H_5	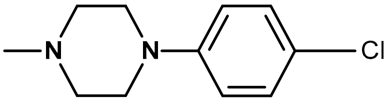
**1F**	CH_3	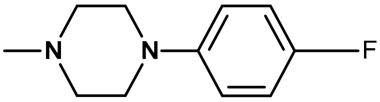	**2F**	C_2H_5	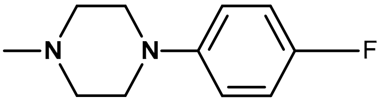
**1G**	CH_3	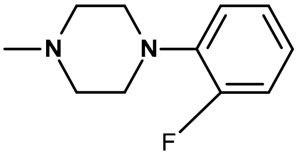	**2G**	C_2H_5	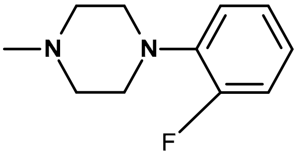

Figure S1. Structure of 1H-pyrollo[3,4-c]pyridine-1,3(2*H*)-dione derivatives previously published discussed in the text (**1A–F,2A–F**). Part I.

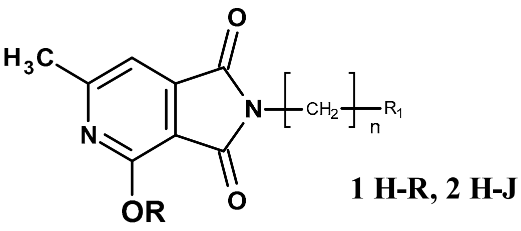

1 H-R, 2 H-J

Comp.	R	n	R1	Comp.	R	n	R1
**1H**	CH_3	3	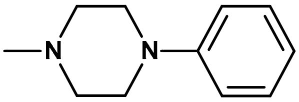	**1Q**	CH_3	4	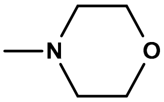
**1I**	CH_3	3	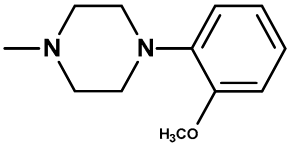	**1P**	CH_3	4	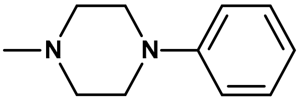
**1J**	CH_3	3	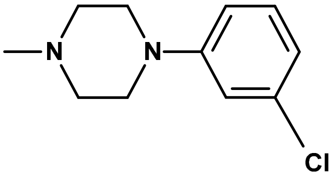	**1R**	CH_3	4	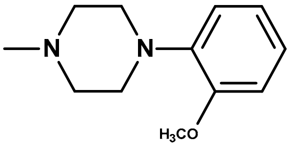
**1K**	CH_3	1	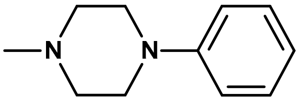	**2H**	C_2H_5	3	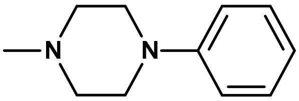
**1L**	CH_3	1	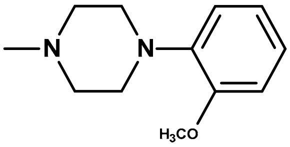	**2I**	C_2H_5	3	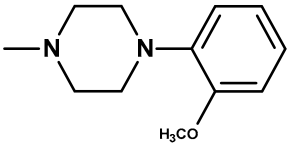
**1M**	CH_3	1	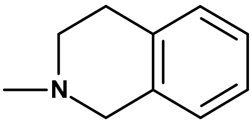	**2J**	C_2H_5	3	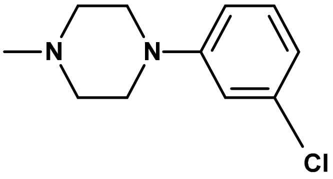
**1N**	CH_3	4	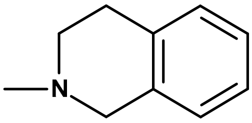				

Figure S2. Structure of 1*H*-pyrollo[3,4-c]pyridine-1,3(2*H*)-dione derivatives previously published discussed in the text(**1H–R,2H–J**).Part II

Table S1. Influence of the compounds investigated on the pain reaction in the "writhing" test in mice.

Compounds	Dose (mg/kg)	Mean no. of writhing ± SEM	ED_{50} (mg/kg)± SEM
**Control**	0	32.2 ± 3.0	
**8**	50	3.8 ± 2,1***	14.5 ± 0.03 (11.15-11.28)
	25	6.2 ± 0.9**	
	12.5	19.8 ± 2.1	
**Control**	0	29.7 ± 30	
**9**	37.5	1.2 ± 0.7****	3.67 ± 0.49 (2.82-4.77)
	18.75	5.0 ± 2.0****	
	9.375	9.8 ± 1.8****	
	4.68	22.0 ± 4.6	
**Control**	0	24.0 ± 2.8	
**10**	100	0.9 ± 0.1****	15.8 ± 0.91 (14.1-17.7)
	50	2.1 ± 0.6****	
	25	7.9 ± 2.1**	
**Control**	0	29.7 ± 3.0	
**11**	50	1.4 ± 0.6 ****	3.25 ± 0.80 (2.01-5.16)
	12.5	4.3 ± 1.1 ****	
	6.25	6.0 ± 2.8 ****	
	3.125	17.7 ± 2.4*	
**Control**	0	24.0 ±2.8	
**12**	100	1.9 ± 0.4****	14.9 ± 2.01 (11.5-19.4)
	50	2.4 ± 0.3****	
	25	9.7 ± 1.1 ****	
	12.5	13.9 ± 2.3	
**Control**		24.0 ± 2.8	
**13**	100	0.8 ± 0.1****	14.8 ± 1.40 (12.4-17.9)
	50	3.8 ± 1.9***	
	25	5.9 ± 0.8**	
	12.5	14.1 ± 1.0	
**14**	100	1.8 ± 0.3****	18.4 ± 1.73 (15.3-22.1)
	50	5.9 ± 1.9***	
	25	9.1 ± 0.8*	
	12.5	15.1 ± 4.2	
**15**	100	1.4 ± 0.4****	19.2 ± 2.14 (14.3-22.7)
	50	2.9 ± 1.9***	
	25	9.4 ± 2.2**	
	12.5	17.2 ± 3.2	
**Control**	0	19.2 ± 3.2	
**ASA**	100	3.2 ± 1.1****	39.15 ± 4.84 (29.1-48.1)
	50	8.5 ± 1.3**	
	30	11.2 ± 2.1	
**Morphine**	10	1.2 ± 0.8****	2.44 ± 0.97 (1.18-5.02)
	3	7.5 ± 2.9**	
	1	16.2 ± 3.51	

Each group consisted of 6-8 animals. **** P < 0.001, ***P < 0.01, **P < 0.02. *P < 0.05.

Table S2. Influence of the compounds investigated on the spontaneous locomotor activity in mice.

Compounds	Dose (mg/kg)	Degree of motor inhibition (%)	Number of impulses ± SEM (30min)	ED_{50} (mg/kg)±SEM
**Control**	0		464 ± 25.9	
**8**	50	61.64****	178 ± 30****	34. 2 ± 8.50 (21.37-54.72)
	25	35.36**	299 ± 59**	
	12.5	32.76**	312 ±26**	
	5	18.1	387 ± 24	
**Control**	0		451 ± 68	
**9**	37.5	67.18***	148 ± 32***	18.8 ± 4.00 (12.5 – 28.2)
	18.75	45.90**	244 ± 45***	
	93.75	42.79*	258 ± 47.4**	
	4.68	22.62	22.0 ± 4.6	
**Control**	0		441 ± 82	
**10**	100	6.8****	142 ± 29****	84.0 ± 5.10 (75 - 95)
	50	55.10***	198 ± 54***	
	25	49.20**	224 ± 45**	
**Control**	0		451 ± 68	
**11**	50	68.74***	141 ± 63 ***	19.7 ± 4.89 (12.3 – 31.5)
	25	55.85**	199.1 ± 48 **	
	12.5	55.43*	201 ± 74*	
	6.25	28.16	324 ± 49*	
**Control**	0		441 ± 82	
**12**	200	76.19****	105 ± 48****	85.0 ± 4.20 (77-93.5)
	100	59.18***	180 ± 41***	
	50	34.46*	289 ± 72 *	
	25	11.79	389 ± 54	
**13**	200	59.64***	178 ± 32***	164.0 ± 28.72 (117-229.6)
	100	32.20**	299 ± 49**	
	50	7.93	406 ± 34	
**14**	200	65.99***	150 ± 48***	98.0 ± 13.26 (75.4 -127.4)
	100	52.38**	210 ± 40**	
	50	3.5*	280 ± 46*	
	25	10.65	394 ± 70	
**15**	200	65.53****	152 ± 34****	89.1 ± 4.46 (80-97.5)
	100	53.96***	203 ± 39***	
	50	42.63*	253 ± 48*	
	25	9.52	399 ± 71	

Each group consisted of 6-8 animals. **** P < 0.001, ***P < 0.01, **P < 0.02, *P < 0.05.

Table S3. Determination of the ability to displace ligands labeled with tritium dihydromorphine [^{3}H-DHM] from the μ-receptor binding sites of the rat cortex.

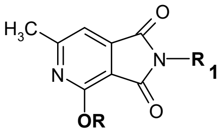

Compounds	R,	R1	Dihydromorphine [^{3}H-DHM] Ki [μM]
**1I**	CH_3,	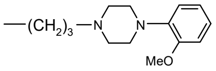	30.8 ± 47.8
**2D**	C_2H_5,	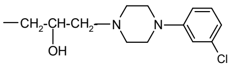	>100
**1J**	CH_3,	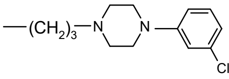	>100
**2J**	C_2H_5,	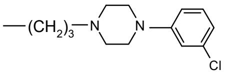	> 100
**1G**	CH_3,	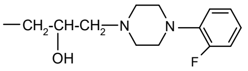	>100
**1M**	CH_3,	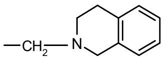	13.8 ± 12.2
